# Dynamic Behavior of Aviation Polymer Composites at Various Weight Fractions of Physical Modifier

**DOI:** 10.3390/ma14226897

**Published:** 2021-11-15

**Authors:** Ewelina Kosicka, Marek Borowiec, Marcin Kowalczuk, Aneta Krzyzak

**Affiliations:** 1 Faculty of Mechanical Engineering, Lublin University of Technology, Nadbystrzycka 36, 20-618 Lublin, Poland; m.borowiec@pollub.pl (M.B.); m.kowalczuk@pollub.pl (M.K.); 2 Faculty of Aeronautics, Military University of Aviation, Dywizjonu 303 No. 25, 08-521 Deblin, Poland; a.krzyzak@law.mil.pl

**Keywords:** composite material, vibrations, resonance zone

## Abstract

The aim of this study was to determine the effect of a selected physical modifier with different granularity and mass percentage on the dynamics of aerospace polymer composites. The tests were carried out on samples made of certified aerospace materials used, among other purposes, for the manufacture of aircraft skin components. The hybrid composites were prepared from L285 resin, H286 hardener, GG 280T carbon fabric in twill 2/2 and alumina (Al_2_O_3_, designated as EA in this work). The manufactured composites contained alumina with grain sizes of F220, F240, F280, F320 and F360. The mass proportion of the modifier in the tested samples was 5% and 15%. The tested specimens, as cantilever beams fixed unilaterally, were subjected to kinematic excitation with defined parameters of amplitude and frequency excitation in the basic resonance zone of the structure. The results, obtained as dynamic responses, are presented in the form of amplitude–frequency characteristics. These relationships clearly indicate the variable nature of composite materials due to modifier density and grain size. The novelty of this study is the investigation of the influence of the alumina properties on system dynamics responses.

## 1. Introduction

Although composite materials, revolutionizing the structural materials market, are found in common manufacturing solutions, the research area surrounding the design of their properties is still open. This is due to both the heterogeneity of their internal structure and the influence of matrix and filler configurations on the resulting composite properties [1,2,3,4]. Moreover, it is difficult to predict the influence of partial physical phenomena that shape the structure’s properties on the final shape of the composite, e.g., mechanical load transfer, thermal and chemical resistance, and abrasive wear resistance [5,6,7]. For these reasons, experimental research has taken on particular importance for both practical and epistemic reasons [8].

A multi-faceted approach to verifying the impact of the modifications applied, whether technological, physical or chemical, is essential, especially in industries that are held to high standards. One example is the aeronautics industry, which requires solutions that meet imposed standards, thus providing quality and safety guarantees [9,10,11]. As a result, proposals for the application of modifications to aerospace materials, in addition to demonstrating the improvement of the selected properties, require the specification of whether these modifications have an effect on other properties, and if there is, to quantitatively and qualitatively specify it.

Despite the numerous requirements and the wide range of research to be carried out, dynamic development in the field of aerospace materials is currently taking place [12,13,14]. The numerous material solutions presented in the literature testify to the constant interest of the scientific community in making a real impact on the occurrence of synergies between the acquisition of knowledge and its practical application [15,16,17,18,19,20,21,22]. The issues related to the modification of polymer matrix composites are new with respect to materials science, although they are extremely extensive due to the intensification of research carried out in scientific centers all over the world [23,24,25,26,27,28]. The considerations presented below represent only a small part of the description of progress in this field, and the works cited are only illustrative of the multifaceted nature of the improvements.

The literature analyzed to date allowed the authors to identify several approaches in the area of modification of composites that have a real impact on the final properties obtained. Among these, the following modifications were observed [1,4,23]:

- The composition of composites by:
  - Modification of the polymer matrix.
  - The use of additives:
    (a) Without applied improvements;
    (b) After chemical improvement of their surface;
    (c) After physical improvement of their surface;
    (d) After biological improvement of their surface;
    (e) After mixed surface improvement.
- Processing through the introduction of additional technological operations resulting in changes:
  - To the physical properties of the composite produced;
  - To the chemical composition of the composite produced;
  - To the biological properties of the composite produced;
  - In the form of a combination of these changes.

The inclusion of an additional modifier component in the composition of composites made on the basis of a matrix of polymeric composition and fabric reinforcement gives rise to hybrid composites. Due to the difficulty of obtaining the sum or resultant properties of the applied modifiers in hybrid composites, it is necessary to conduct research to determine the influence of the presence of additives on the properties of the composite. Examples of modifications to improve strength, ablation or tribological properties, among other things, can be found in the literature. Examples of these are shown in Table 1.

The examples of modifications mentioned above signal only the areas of changes in the properties of composites, showing at the same time the appropriateness of the implemented actions due to the wide range of improvements [29,30,31,32,33,34,35,36,37,38]. Undoubtedly, the research carried out to date in the area of the influence of the composition of polymeric composites on their properties has assisted in the development of their understanding, at the same time providing the foundations for the process of inference or the stage of conducting further changes. These, necessitated by aspects of cost optimization, safety improvement or environmental protection, have resulted in an intensification of the actions undertaken.

The dynamics of ongoing research in the field of composite materials is reflected in numerous papers [39,40,41,42,43,44,45]. The results published to date repeatedly mention the use of carbon fibers as reinforcement for polymer composites. Carbon fibers, obtained in particular by pyrolysis of polyacrylonitrile, are characterized by thermal and chemical resistance, good electrical and thermal conductivity, and low density [46]. In addition, the ability of the fibers to dampen vibrations has also been indicated. These properties greatly influence the final use of carbon fibers for composite applications globally. It should be stressed that the differences in the properties obtained from such materials are closely related to the arrangement of the fibers, the number of layers used or the way in which they are arranged.

As already mentioned, the modifications carried out to the composition of composites affect their properties, including dynamic ones. It is, therefore, necessary to determine the dynamic characteristics of materials, especially if the components made from them are exposed to the negative consequences of the phenomenon of resonance, as is the case, for example, in the area of aircraft structures.

One of the examples presented in the literature, and related to the research of amplitude–frequency characteristics in aviation, is the PZL SW-4 helicopter described in [44]. As part of the study, the authors recorded the frequency spectrum taken from the vertical stabilizer in a helicopter flying horizontally at a constant speed of 200 km/h at an altitude of 1000 m. The measurements revealed a range of frequencies and amplitudes that enabled the identification of important design variables. The results showed that during helicopter flight, noticeable vibration levels occurred at frequencies of 22, 30 and 45 Hz. There are, therefore, sensitive frequencies of the aircraft structure, so research into the changes caused by the introduced modifications to the material composition is appropriate [37,38]. Modifications to material structures open up a wide field of analysis. Dynamic behavior has been investigated in the context of nonlinear parametric resonance [39], where the authors investigated the cubic nonlinear response of beams subjected to a spring force in longitudinal direction. The nonlinear vibrations of beams were also analyzed in [40]. The authors discussed the influence of different support types on free nonlinear vibration frequency. Nonlinear behavior is significant especially for the complex structures of composite cantilever beams. It is worth investigating the influence the composite plies layout on their dynamic behavior. The authors of paper [41] analyzed the fibers sequences of composite materials on the dynamic responses and the energy harvesting efficiency by applied the piezoelectric components. Moreover, the piezoelectric structures are often applied for vibration controls the composite structures as authors discussed in paper [42].

## 2. Experimental Investigation

Material produced in the laboratory of the Air Force Military Academy in Dęblin was tested. It was a polymer composite with high static and dynamic strength, the matrix of which was made of L285 resin approved by the German Federal Aviation Authority. Due to its properties, this resin is widely used in aviation and model making. Since the L 285 laminating resin was certified in 1985, it has been used by many aircraft and glider manufacturers and, due to its very high physiological tolerance, it is now the most widely used system in the aerospace industry [43]. Materials with the described properties are aeronautically certified. The test plan included the use of a dedicated H286 hardener for the selected resin (see in [43]).

In addition, GG 280T carbon fiber fabric with a 2/2 twill weave was used to create the composite. Eight layers of fabric were arranged in each panel in fiber orientation [0/45/90/135/0/45/90/135]. The composite structure presented is a representative example of an aerospace structural material and has already been used in this application.

The authors, on the basis of their knowledge of the literature as well as studies conducted to date devoted to the influence of a physical friction modifier in the form of alumina (aluminium trioxide, Al_2_O_3_, hereafter denoted as EA) on selected mechanical and tribological properties, applied this modifier to prepare the studied composites. The fabric and powder reinforcement resulted in a hybrid composite. The decision to conduct research on composites with specific mass percentages of alumina was based on the results obtained previously, presented in previous works [37], which allowed us to optimize selected mechanical properties and to show comparisons for content levels of 5 and 15%. The study also includes reference samples that were manufactured using only epoxy resin, hardener and carbon fabric.

The alumina used in this study had different grain sizes determined according to FEPA 42-2:2006 standards [44], and the corresponding grain sizes are shown in Table 2.

The composite material was manufactured by hand lamination. The prepared fabric layers were successively impregnated with an alumina resin of different mass percentages and grain sizes, and finally compressed using a PDM-50S Mecamaq hydraulic press at 2.5 MPa. Environmental conditions did not affect the polymer composites; they were not subjected to external factors such as changes in temperature, humidity or radiation. The composites were stored under laboratory conditions without exposure to sunlight.

In the absence of a standard defining the dimensions of the test specimens, beams with an active length of 200 mm, a width of 10 mm and a thickness of 2.4 mm were used, as in previous studies [37,38]. Test specimens with the desired dimensions were prepared from composite panels by shaping them using an abrasive water jet cutting process. Ten specimens were prepared for each mass percentage and grain size of the alumina, with the following dimensions: length—200 mm; width—10 mm. Table 3 shows the determination of the composites tested.

Performing the subsequent part of the research experiment, in which the samples were subjected to kinematic forcing with defined parameters of amplitude and frequency in the area of the basic resonance of the material, required knowledge as to the density of the composites produced. This was determined on the basis of density measurements using the XSE205DU/M precision laboratory balance (Mettler Toledo, Zurich, Switzerland). The density determination of the composites was carried out in accordance with ISO 1183-1:2019 [45].

## 3. Measurement Approach

**Figure 1:**
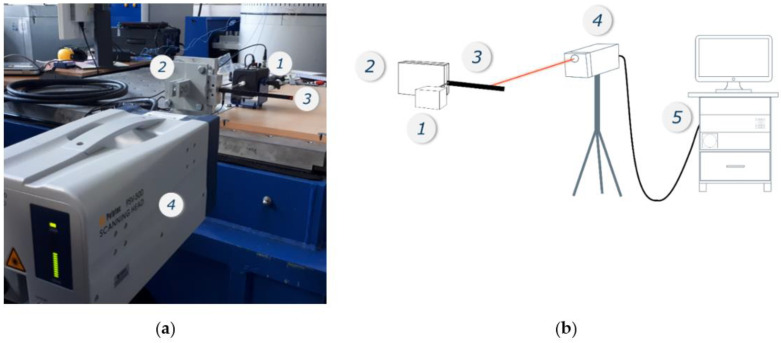
View of the test stand (**a**); schematic diagram of the laser vibrometer components (**b**). Parts of the measurement system: electro dynamic shaker (**1**), support of the beam (**2**), sample (**3**), PSV-500 scanning head (**4**), and OFV-500 controller (**5**).

Dynamic analysis of the individual composite samples was carried out using a non-contact vibration measurement technique. This approach eliminates the interference that occurs with measurements using acceleration sensors. For susceptible specimens with relatively low stiffness, the mass of the sensors significantly changes the inertia of the system during vibration. A Polytec -500-3D laser vibrometer from EC Test Systems was used for the measurements. The principle of operation of this type of setup is a comparison of the laser beam reflected from the object being tested with the reference signal of the photo detector to which the signal is returned. This is carried out based on the Doppler effect, where vibrations caused by an electrodynamic inductor cause changes in the frequency of waves reaching the receiver. As shown in Figure 1, a SmartShaker Mini K2007E01 electrodynamic inductor (1) was used to apply the excitation near the handle (2) directly to the specimens (3). To capture the dynamic response of the sample, a single laser head (scanning head) was used (4). The signal obtained during the measurement was recorded as a function of the forcing frequency. Using the measuring apparatus software (5), the frequency range was determined, including the first three flexural vibration modes of the tested composite specimens.

The standard calibration of the vibrometer has an accuracy of 0.1 µm/s. In the applied measuring system, calibration was performed for 2D and 3D alignments at double the recommended number of points [46].

## 4. Results and Discussion

**Figure 2:**
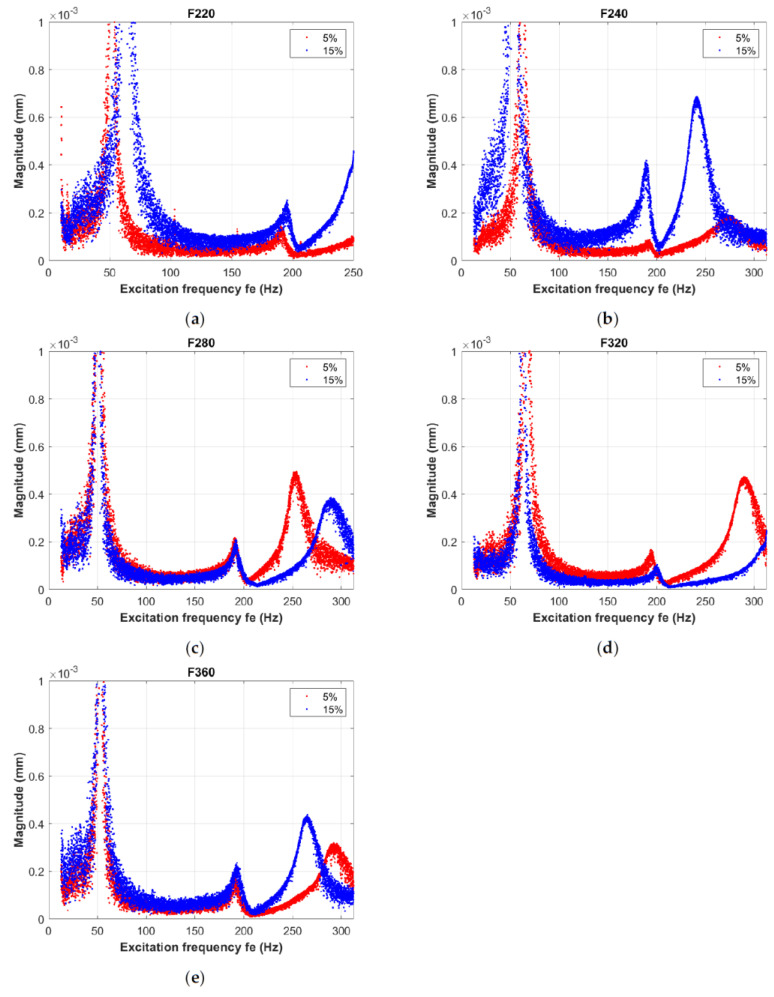
The amplitude–frequency responses of the beams measured by vibrometer scanning head PSV-500 for 5% and 15% concentrations of modifier in the cases of samples with coarse-grained alumina F220 (**a**), F240 (**b**), F280 (**c**), F320 (**d**), and (**e**) F360, respectively.

Measurements were performed under laboratory conditions for a set of specimens in the form of beams, as described in Section 2. The study was designed to determine the effect of the modifier used on dynamic responses at varying granularities. The alumina modifier was used in two variants—5% and 15% by weight, respectively—and five grain sizes (F220, F240, F280, F320 and F360). The results obtained and discussed here represent a continuation of our previous investigations, reported in [37,38]. Initially, the dynamic behavior of composite materials was analyzed with respect to different positioning of the fabric configuration sets in cantilever beams during dynamic tests [37]. These experiments included the composite structure for one chosen grain of alumina (F280), and for one weight fraction of physical modifier (10%). In the second paper [38], we broadened our investigations to three values of weight fraction of physical modifier with a single grain value (F280). To obtain meaningful results, amplitude–frequency measurements were carried out on ten sets of samples for each series with respect to mass proportion and grain size. This section presents the results averaged over the samples received. Figure 2 shows graphs grouped by modifier granularity and compares the vibration behavior of the system at both modifier mass percentages. In the sample set for the lowest granularity (F220), the response of the composite at a concentration of 15% was shifted, compared to the results obtained for a concentration of 5%, towards higher frequencies across the forcing range by approximately 15 Hz. Particularly noticeable is the shift in the fundamental resonance, which could indicate that an increase in the mass proportion of the modifier results in an increase in the effective stiffness of the composite. Thus, in the next tested set, for grain size F240 (Figure 2b), the changes were much more noticeable, but were the opposite of those depicted in Figure 2a. The fundamental resonance underwent a shift of about 20 Hz towards the lower frequencies with a modifier concentration of 15% compared to 5%. In addition, the system was able to achieve significantly higher relative amplitudes when operating in the second and third forms of vibration with a modifier mass percentage of 15%. In the second resonance, noted at 190 Hz, and in the third at 240–275 Hz, the response amplitudes were four times larger for a concentration of 15% compared to a concentration of 5%, and there was a shift of the resonance peak towards the lower frequencies in the last of 35 Hz. These characteristics clearly show that the composite with a 15% modifier concentration by mass had a significantly lower effective material stiffness in the whole investigated region of the forcing frequency. On the other hand, the dynamics of the material using modifier with the F280 and F320 grain sizes converged in the region of the first and second resonances. Here, changes in the amplitude response occurred for the third form of vibration. Operating at frequencies above 200 Hz caused the resonance peak to shift to the left by about 50 Hz for the material with a 5% modifier concentration compared to the composite with a 15% modifier concentration (Figure 2c).

**Figure 3:**
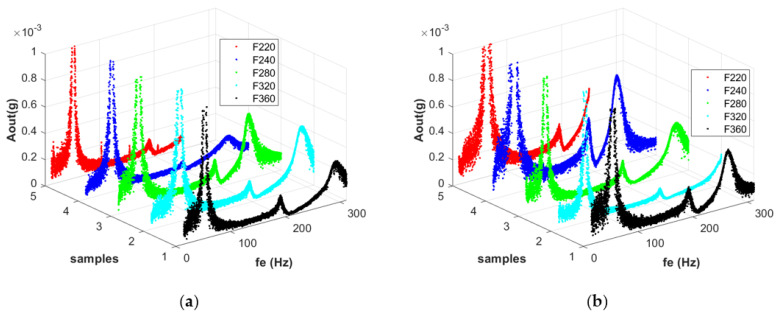
Dynamic response of the beams for the analyzed grains at a concentration of 5% of modifier (**a**,**b**) a concentration of 15% of modifier for all coarse-grained alumina.

At the same time, the occurrence of the third form of vibration at much higher frequency values is noticeable in the case of the composite containing modifier with a grain size of F320 (Figure 2d—blue dots) compared to the F280 variant (Figure 2c—blue dots). From the observed shift of the resonance peaks to the right in Figure 3 compared to the characteristics in Figure 2b, it can be concluded that a material with a higher grain size at relatively high vibration frequencies demonstrates an increased effective stiffness regardless of the modifier mass percentage. The last of the granularity variants tested, F360 (Figure 2e), again demonstrated a response character compatible with the results for the F220 and F240 granularities. Here, a modifier concentration of 15% caused a shift of the third resonance to the left compared to the response for a concentration of 5%, as shown in Figure 2b. Thus, increased granularity reduced the effective stiffness.

**Figure 4:**
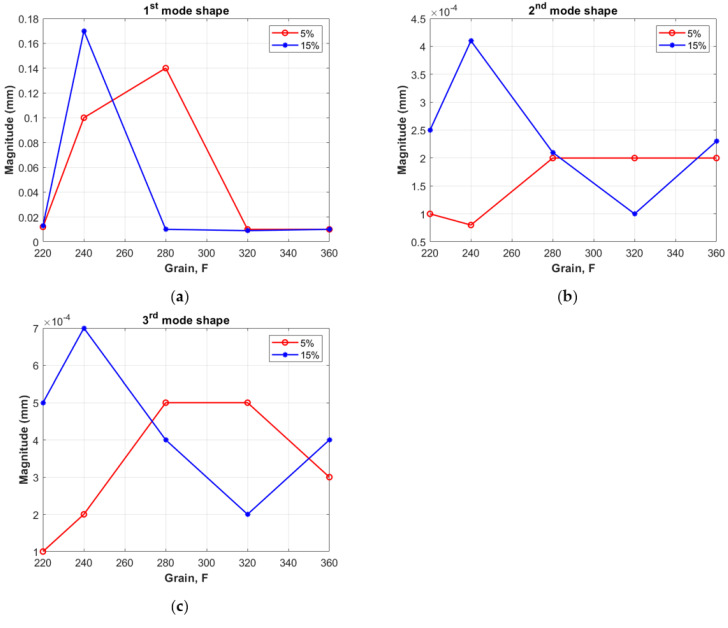
The magnitude of the amplitude responses of the beams via grains of the modifier. Results for the first mode shape (**a**), the second mode shape (**b**), and the third mode shape (**c**), respectively.

Figure 3 shows the characteristics grouped according to the concentration of modifier by mass to show the influence of the granularities tested on the composite dynamics. From Figure 3a,b, the relationships shown in Figure 4a–c emerge. Materials with an alumina additive at concentrations of 5% and 15% behave similarly in the region of the first resonance, which is in the range 50–70 Hz. The values of the amplitudes increase with granularity F240, after which they decrease (Figure 4a).

However, at higher frequencies, where the second and third forms of vibration appear, 170–320 Hz, the different natures of the studied composites become apparent. With increasing granularity, starting at F220, the amplitude responses of the system with the 15% modifier increase (F240), then decrease (F280, F320) and increase again (F360), occupying a position averaged over the first four granularities (Figure 4b blue lines). If, on the other hand, a modifier concentration of 5% is used, the increase in grain size results in a tendency for the vibration amplitudes to increase up to F280, after which the system stabilizes in the second mode, and then decreases again in the case of the third form of vibration (Figure 4b,c red lines). The graphs illustrate how variable the nature of the composite material is. The characteristics of the changes in amplitudes are indicative of the non-linear dynamic responses of the beams, assuming a variable of the so-called ‘effective stiffness’ of the material.

## 5. Conclusions

In this study, we analyzed the influence of two parameters of a modifier that was a component of the tested material on the vibration behavior of composite beams in the frequency range covering the first three forms of vibration. These were the mass percentage of the modifier in the composite structure and its granularity. The first parameter was narrowed down to two values, while the second included five grain sizes related to the grain size of the alumina. Experimental studies showed that the dynamics of the structures studied varied with the proportion of modifier, with different granularity. It turns out that the operation of the beams at the first form of vibration behaves differently to the higher mods. 

The results, obtained as the amplitudes of beam end displacement, provide information about the so-called effective stiffness of the system. In interpreting the results, reference was made to the relationship represented by displacement and stiffness. It is well known from materials mechanics that when a constant amplitude is enforced on a system, materials with a relatively lower stiffness reach higher amplitudes of vibration in response. This apparent relationship was observed when the individual samples were tested. While relatively similar responses can be expected in the first resonance region, at higher vibration frequencies the amplitude–frequency characteristics are not unambiguous. Therefore, on the basis of the analyses carried out, it is possible to optimize the choice of the modifier mass percentage together with its granularity, where the objective function will be to achieve relatively minimal vibration amplitudes in the system. The knowledge of the choice of these parameters makes it possible to modify the dynamics of structures by changing their natural frequencies, which in technical applications is of great importance due to the need for composite materials to work in a safe range of frequencies.

## Figures and Tables

**Table 1:** Examples of application of physical modifiers affecting the change of selected properties of composites.

Modified Properties	Modifier	Specification
mechanical	18-acyl-dopamine	The results of the research conducted showed that the addition of 18-acyl-dopamine can be used as an effective modifier of composites based on a matrix of high-density polyethylene additionally reinforced with bamboo powder. When the modifier increased, the hardness of the composite deteriorated, while strength and stiffness improved. SEM analysis showed that the bond between the bamboo powder and the plastic matrix was strongest when the modifier 18-acyl-dopamine was 1.25% by mass [20].
heat	aerogel	An epoxy resin filled with aerogel particles was tested for the effect of resin viscosity on pore infiltration and density of the resulting composites. Furthermore, the effects of aerogel content and particle size on the thermal conductivity and compressive properties of epoxy composites are presented. The study was conducted on a resin that is a mixture of bisphenol-A epoxy resin and epichlorohydrin-formaldehyde-phenol polymer, and a cycloaliphatic amine-based hardener was used as the curing agent. The introduction of silica aerogel particles into the resin led to a significant reduction in both the density of the resin and its thermal conductivity [21].
ablative	PCM (phase-change material)	The use of an additive in the form of PCM (Phase Change Materials) causes some of the energy supplied to the system to be ‘consumed’ by the phase change energy of the PCM material. This improves the thermo-protective properties of the composite—it significantly reduces the ablative weight loss by about 30% and the temperature on the back surface of the tested composite by about 50% [22].

**Table 2:** Information on the grain size of the alumina.

Indication of the grain size of the alumina used	F220	F240	F280	F320	F360
Grain size according to FEPA 42-2:2006 [µm]	53	44.5	36.5	29.2	22.8

**Table 3:** Composition and designation of manufactured composites.

Epoxy Resin	Hardening Agent	Carbon Fabric	Physical Modifier	Granularity	Mass Percentage of Modifier	Designation of the Composite
L285	H286	GG 280T	-	-	-	GG 280T/EA/0/0
			ALUMINA (EA)	F220	5%	GG 280T/EA/F220/5
					15%	GG 280T/EA/F220/15
				F240	5%	GG 280T/EA/F240/5
					15%	GG 280T/EA/F240/15
				F280	5%	GG 280T/EA/F280/5
					15%	GG 280T/EA/F280/15
				F320	5%	GG 280T/EA/F320/5
					15%	GG 280T/EA/F320/15
				F360	5%	GG 280T/EA/F360/5
					15%	GG 280T/EA/F360/15

## Data Availability

Not applicable.
